# The Plans and Purposes of the Johns Hopkins Hospital

**Published:** 1889-09-07

**Authors:** John S. Billings

**Affiliations:** Surgeon, U.S. Army


### The Plans and Purposes of the Johns Hopkins Hospital.*

By John S. Billings, M.D., Surgeon U. S. Army.

(Concluded from page 346)

### IV.—THE HOSPITAL AND MEDICAL EDUCATION.

On the philanthropic, social, and religious aspects of this great trust I do not propose to touch; but I wish to say a very few words of the hopes and wishes of scientific men and physicians with regard to it. From the time of the first announcement of the Hopkins bequests to the present, these men, all over the world, have been keenly interested in the plans and methods adopted in carrying them out. Whenever and wherever the problems of higher medical education have been discussed within the last ten years, there has been speculation as to the probable course of the Johns Hopkins medical department, and the influence it would have upon the standard. I may even say that some of this influence has been exerted in advance—has been discounted, as it were—for the plans of this hospital have stimulated changes in some of our best medical schools, and have been copied with more or less modification in some of our latest hospitals.

What is it, then, that the physicians want? Is it more physicians, more family practitioners, more surgeons, more specialists? Not at all. They know very well that there is no danger that the supply will not be equal to the demand; when they become overburdened with practice they do not at present find it difficult to obtain assistants; they have no fear lest the seventy or eighty medical schools of this country should fail to produce a sufficient number of medical practitioners to meet the wants of our increasing population; and they know also that medical schools of Great Britain and Germany are sending to us quite as much of their product as we can conveniently dispose of. They hope that the Hopkins medical school and hospital will do two things. The first is, that it will demand of those who propose to become its students evidence that they have a sound basis of preliminary education before they commence, and that its standard in this respect shall be little below that of the requirements for granting the degree of bachelor of arts in the university. It is hoped that the men thus selected will go through a carefully-graded course of study, including actual work in properly-fitted laboratories, and that after this they will be brought into contact with the sick, and thus obtain practical experience of the duties and responsibilities of the practitioner of medicine before they offer their services as such to the public.

So much our physicians desire of every medical school, for the sake of the honour and dignity of the profession, and for the good of the public, and they desire especially that this school shall form an example to which they can point as showing how medical education should be conducted, and what should be required of the candidate for the degree of doctor of medicine.

The very general interest in the combined Hopkins trusts felt by physicians and scientific men, not only of this country but of the whole civilised world, is largely due to the belief that the relations which will here exist and be maintained between the university as a whole and its medical department, of which this hospital is to be an important part, will be close and intimate, so that the true university spirit will pervade, stimulate, and encourage the hospital work. In this country medical schools have either had no connection with universities properly so called, or the connection has been slight and nominal, such as depends upon the formal conferring of medical degrees by the university. Here, however, through the influence of the biological department, there are secured common interests and mutual influence, and it is hoped, therefore, that the necessary details of technological instruction will be arranged in accordance with, and subordinate to, the broad principles of scientific culture upon which this university is organised.

It is because it is believed that this will be the case that there is a widespread hope and expectation that these combined institutions will endeavour to produce investigators as well as practitioners, to give to the world men who can not only sail by the old charts, but who can make new and better ones for the use of others. This can only be done where the professors and teachers are themselves seeking to increase knowledge, and doing this for the sake of the knowledge itself; and hence it is supposed that from this hospital will issue papers and reports giving accounts of advances in, and of new methods of acquiring knowledge, obtained in its wards and laboratories, and that thus all scientific men and all physicians shall share in the benefits of the work actually done within these walls. But however interesting and valuable this work may be in itself, it is of secondary importance to the future of science and medicine, and to the world at large, in comparison with the production of trained investigators, full of enthusiasm, and imbued with the spirit of scientific research, who will spread the influence of such training far and wide. It is to young men thus fitted for the work that we look for the solution of some of the myriad problems which now confront the biologist and the physician.

Do I seem to ask too much? To be too sanguine as to what human thought, and study, and skill may accomplish? To forget that there is one event unto all; that the shadow of pain and death comes on the wise man as on the fool? I have two answers. As surely as our improved methods of prevention and treatment, based on the advances in knowledge of the last fifty years, have already extended the average duration of life in civilised countries nearly five years, have prolonged thousands of useful and productive lives, and have done away with the indescribable agonies of the preanæsthetic period, so surely we are on the verge of still greater advances, especially in the prevention of infectious and contagious disease, in the resources of surgery against deformities and morbid growths, and in the mitigation of suffering due to causes which cannot be wholly removed. But the second answer is more important, and it is this: It is our duty to try to increase and diffuse knowledge according to the means and opportunities which we have, and not to rest idle because we cannot certainly foresee that we shall reap where we have strewn. "It is not incumbent on thee to finish the work, but thou must not therefore depart from it," says the Talmud, and "Of him to whom much is given much shall be required," says the Scripture.

To you, the officers of this institution, and to you, men and women of Baltimore, there is now given the opportunity of giving powerful aid in this increase and diffusion of knowledge of the laws of human life, disease, and death. Surely, those who are working in the wards and laboratories of the hospital and university will do their best; surely, also, the citizens of this great city of a great nation, which at no distant day will take the lead in scientific work, will encourage, sustain, and sympathise with these workers. I would have this hospital become famous, not for fame's sake, but because this will be evidence of the good work which has been done in it. But we must not be impatient. There are difficulties to be overcome, delays which must be submitted to. We cannot at once have the medical school which is essential to the plan which I have sketched; but there is plenty to do for the present, and I am certain that in time all these present obstacles to full development will be happily overcome.

Success in this, as in all other enterprises in this world, is to be obtained by unselfish work for the good of others, by wise counsel, by co-operation, and by persistent effort.

A hospital is a living organism, made up of many different parts, having different functions, but all these must be in due proportion and relation to each other, and to the environment, to produce the desired general results. The stream of life which runs through it is incessantly changing. Patients, and nurses, and doctors come and go. To-day it has to deal with the results of an epidemic, to-morrow with those of an explosion or a fire. The reputation of its physicians or surgeons attracts those suffering from a particular

* An address delivered at the opening of the hospital, May 7, 1889.

form of disease, and as the one changes so do the others. Its work is never done; its equipment is never complete. It is always in need of new means of diagnosis, of new instruments and medicines; it is to try all things and hold fast to that which is good.

"Et quoniam variant morbi, variabimus artes."

It has been said that "hospitals are in some sort the measure of the civilisation of a people," but a hospital of this kind should be more than an index. It should be an active force in the community in which it is placed. When the mediæval priest established in each great city in France a Hôtel Dieu, a place for God's hospitality, it was in the interests of charity as he understood it, including both the helping of the sick poor and the affording to those who were neither sick nor poor an opportunity and a stimulus to help their fellow-men; and doubtless the cause of humanity and religion was advanced more by the effect on the givers than on the receivers. It is the old lesson so often expounded, apparently so simple and yet so hard to learn, that true happiness lies in helping others; that it is more blessed to give than to receive.

In some respects we of to-day have a much wider outlook than the men of a thousand years ago. This hospital is designed, as I have told you, to advance medical science as well as to give relief to the sick poor, but the fundamental motive is the same—to help others.

We have here the beginning of an institution which shall endure long after the speakers and the audience of to-day shall have finished their life-work and have passed away. Founded in the interests of suffering humanity, intimately connected with a great university, amply provided with what is at present known to be essential to its work, we have every reason to predict for it a long and prosperous career, with steadily progressing improvement in its organisation and methods, and enlargement of its activity and influence.

Let us hope that before the last sands have run out from beneath the feet of the years of the nineteenth century it will have become a model of its kind, and that upon the centennial of its anniversary it will be a hospital which shall still compare favourably, not only in structure and arrangement, but also in results achieved, with any other institution of like character in existence.

